# The Clinicopathologic Features and Treatment of 607 Hindgut Neuroendocrine Tumor (NET) Patients at a Single Institution

**DOI:** 10.1097/MD.0000000000003534

**Published:** 2016-05-13

**Authors:** Seung Tae Kim, Sang Yun Ha, Jeeyun Lee, Sung No Hong, Dong Kyung Chang, Young Ho Kim, Yoon Ah Park, Jung Wook Huh, Yong Beom Cho, Seong Hyeon Yun, Woo Yong Lee, Hee Cheol Kim, Young Suk Park

**Affiliations:** From the Division of Hematology-Oncology (STK, JL, YSP), Department of Medicine; Department of Pathology & Translational Genomics (SYH); Division of Gastroenterology (SNH, DKC, YHK), Department of Medicine; and Department of Surgery (YAP, JWH, YBC, SHY, WYL, HCK), Samsung Medical Center, Sungkyunkwan University School of Medicine, Seoul, Korea.

## Abstract

The clinicopathologic features of hindgut neuroendocrine tumor (NET) as well as the treatment outcomes are not well known. There are currently no published data on treatment outcomes for patients with metastatic hindgut NET. The aim of this study was to conduct a comprehensive analysis of clinicopathologic features, treatments and survival in hindgut NET patients. Among patients who were pathologically diagnosed with hindgut NET at Samsung Medical Center between March 2001 and February 2015, 607 were analyzed in this study. Hindgut NETs were defined as NETs that originated from the transverse and distal colon, rectum, and anus. Primary sites included 81 colon (13.3%) and 526 rectum (86.7%). According to the WHO classification, 578 patients (95.2%) had grade 1 NETs, 17 (2.8%) grade 2 NETs, and 12 (2.0%) had neuroendocrine carcinoma (NEC). Forty-two patients (6.9%) had extensive disease, while the majority (93.1%, 565 patients) only exhibited localized disease. The 5- and 10-year survival rates of 565 localized NET patients were 98.1% and 95.3%, respectively. The median OS in 42 patients with extensive disease was 24.8 months (95% CI, 10.7–38.8). Among 565 patients with localized disease, the majority (484 patients, 85.7%) were treated with endoscopic procedure by gastroenterologists. For 42 patients with extensive disease, 17 patients were managed by supportive care, 3 by concurrent chemoradiotherapy (CCRT), and 22 by systemic therapy. Among these 22 patients, 12 patients received only first-line therapy, 8 had second-line, and only 2 patients had third-line therapy. As first-line chemotherapy, the most commonly used regimens were etoposide plus cisplatin (N = 7) and long acting octreotide (N = 7). During treatment courses, the most commonly used regimen was long-acting octreotide. The median OS in 22 metastatic NET patients receiving systemic therapy was 19.3 months (95% CI, 3.2–35.3). Multivariate analysis in all 607 hindgut NETs patients suggested that the extent and the primary site of disease were significant independent prognostic factors for long term survival. This analysis provides useful information about the clinicopathologic features, treatments and survival outcomes for hindgut NET patients.

## INTRODUCTION

Neuroendocrine tumors (NETs) are composed of a heterogeneous group of malignancies derived from neuroendocrine cell compartments, with roles in both the endocrine and the nervous system. The majority of NETs are gastroenteropancreatic (GEP) in origin, arising in the foregut, midgut, or hindgut.^1^ Within the gastrointestinal tract, most carcinoid tumors occur in the small intestine (41.8%), rectum (27.4%), and stomach (8.7%). The incidence of gastric and rectal carcinoid has increased remarkably, with the most common site being the small intestine, as opposed to the appendix in the past.^2^ Anatomically, the distal colon and rectum originate from the embryonic hindgut.^3^ The racial distribution of hindgut NETs differs significantly from that of NETs of other primary sites, with higher rates observed in blacks and Asians compared with whites.^4^ In a previous Korean gastroenteropancreatic (GEP)-NET registry study, most GEP-NETs were found in the rectum or the stomach, and the most common site was the rectum.^5^

Hindgut NETs are mostly discovered incidentally during routine surveillance endoscopies. Other symptoms include rectal bleeding, pain, and change in bowel habits.^6,7^ Approximately 50% of hindgut NET patients are asymptomatic.^7^ Hindgut ENTs are rarely associated with a hormonal syndrome such as flushing or diarrhea, even in the metastatic stage.^8^ These data are based on Western populations. However, as compared with foregut and midgut NETs, the clinicopathologic features and treatments of hindgut are not well known. Especially, there are currently no published data on treatment outcomes for patients with metastatic hindgut. Given the lack of high-level evidence supporting any type of treatment for metastatic hindgut NETs, an expert panel recommends that clinical trials be considered for all lines of therapy. Consequently, recommendations must be extrapolated from trials of other GEP-NETs.^8^

The aim of this study was to conduct a comprehensive analysis of clinicopathologic features, treatments, and survival outcomes in hindgut NET patients in order to produce fundamental data for future research on these tumors. Simultaneously, we reviewed the details of chemotherapies and survival outcomes for metastatic hindgut NET patients.

## METHODS

### Patients and Tumor Grade

We analyzed the clinicopathologic features, treatments, and survival for hindgut patients who were diagnosed at Samsung Medical Center (SMC) between March 2001 and February 2015. Hindgut NETs included those of the transverse and distal colon, rectum, and anus. Medical records and slides were reviewed for all these patients. The following clinicopathological and treatment variables were analyzed as follows; gender, age, primary site, disease status (localized disease vs extensive disease), metastatic sites, hormonal symptom, date of treatments, types of treatments, surgical or endoscopic reports, chemotherapy sheets, and follow-up).

Tumors of all patients were reviewed and classified by grade according to the 2010 WHO classification. The 2010 classification was performed based on the grading of mitosis or Ki67 labeling index. Mitosis was reported as G1 (<2/10 HPF), G2 (2–20/10 HPF), and G3 (>20/10 HPF). The Ki67 labeling index was G1(≤2%), G2(3–20%), and G3(>20%).

### Statistical Analyses

Descriptive statistics for patient clinicopathologic features were reported as proportions and medians. Data were also presented as number (%) for categorical variables. Overall survival (OS) was defined as the time from the first treatment to the date of death, respectively. Kaplan–Meier estimates were used in the analysis of all time to event variables, and the 95% confidence interval (CI) for the median time to event was computed.

### Ethics Statement

The institutional review board of the Samsung Medical Center (SMC) approved the process of this study. The methods in this study were carried out in accordance with the approved guidelines by SMC and all protocols were approved by the ethics committees of SMC.

## RESULTS

### Patient Characteristics

Among patients who were pathologically diagnosed with hindgut NETS in Samsung Medical Center between March 2001 and February 2015, 607 were analyzed in this study. The baseline characteristics of these 607 patients are listed in Table 1. The median age of the patients was 51.0 years (range 18–80) and the male-to-female ratio was 1.92. Primary sites included 81 colon (13.3%) and 526 rectum (86.7%). According to WHO classification, 578 patients (95.2%) had grade 1 NETs, 17 (2.8%) grade 2 NETs, and 12 (2.0%) had neuroendocrine carcinoma (NEC). Forty-two patients (6.9%) had extensive disease and the majority (93.1%, 565 patients) had only localized disease. Among 42 patients with extensive disease, 31 patients had liver metastases. Hormonal symptoms such as diarrhea, facial flushing, or sweating were reported in only 3 patients (0.5%).

**TABLE 1:**
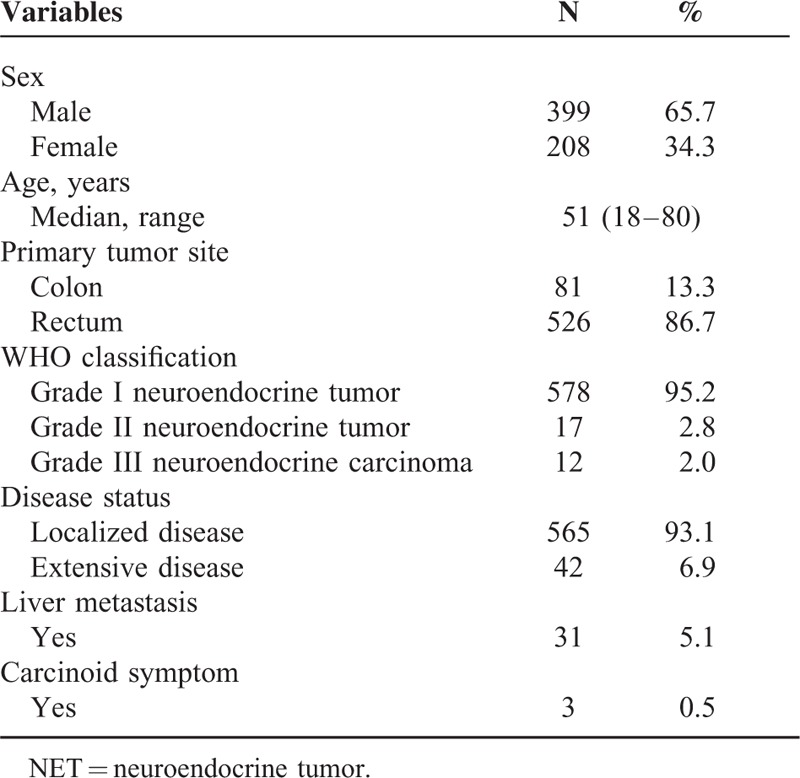
Baseline Characteristics of 607 Hindgut Neuroendocrine Tumor (NET) Patients

### Treatments in 607 Hindgut NETs Patients

**FIGURE 1:**
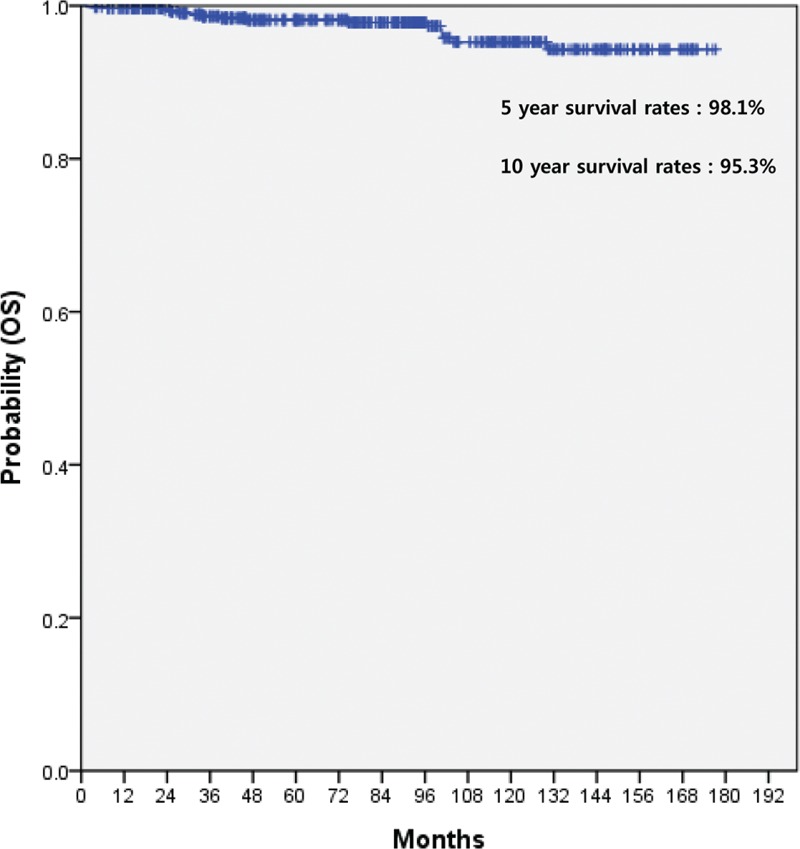
Kaplan–Meier curve for overall survival (OS) in 565 localized hindgut NET patients. NET = neuroendocrine tumor, OS = overall survival.

**FIGURE 2:**
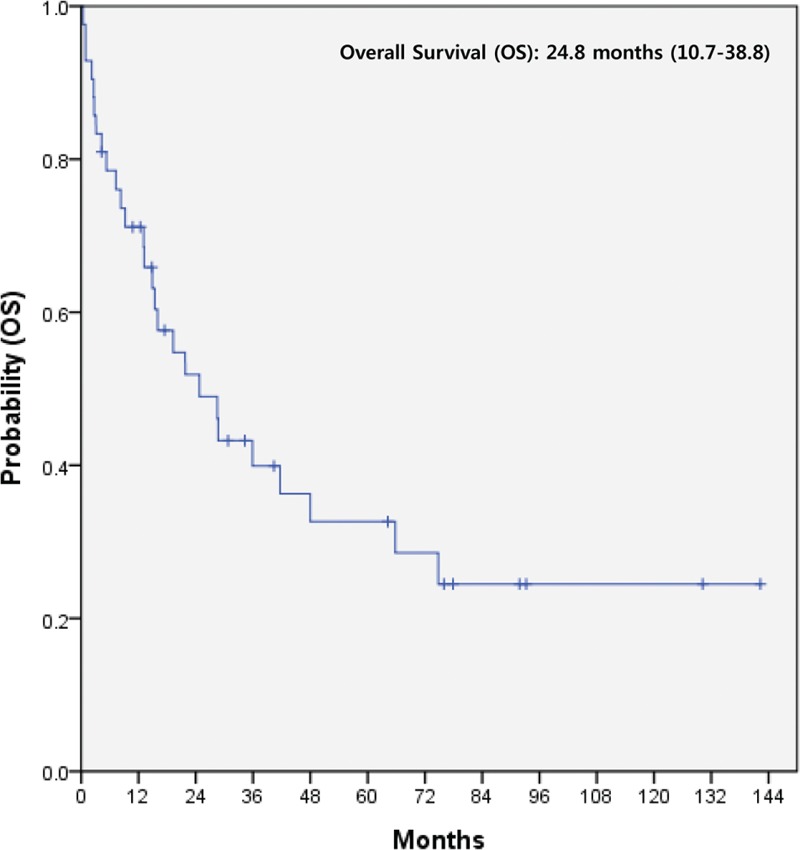
Kaplan–Meier curve of overall survival (OS) in 42 metastatic hindgut NET patients. NET = neuroendocrine tumor, OS = overall survival.

Table 2 summarizes the treatment patterns conducted in all 607 patients according to disease extent (localized disease vs extensive disease). Among 565 patients with localized hindgut NET, the majority (484 patients, 85.7%) were treated with endoscopic procedure by gastroendoscopists. Fifty-five patients (9.7%) received trans-anal microscopic surgery and 26 (4.6%) laparoscopic surgery. The median overall survival (OS) was not reached in 565 patients with localized disease (Figure 1). The 5- and 10-year survival rates of 565 localized NET patients were 98.1% and 95.3%, respectively. For 42 patients with extensive disease, 17 patients were managed by only supportive care, 3 by concurrent chemoradiotherapy (CCRT), and 22 by systemic therapies such as cytotoxic chemotherapy, molecular targeted agents, and somatostatin analogs. For 42 patients, the median overall survival (OS) was 24.8 months (95% CI, 10.7–38.8) (Figure 2). Univariate analysis in all 607 patients revealed that a decreased OS was significant associated with the following variables: extensive disease, colon as primary site, WHO grade I and II, and liver metastasis. In multivariate analysis, extensive disease (HR 23.898, 95% CI 8.309–68.735, *P* = 0.001) and colon as primary site (HR 2.800, 95% CI 1.235–6.349, *P* = 0.014) were independent prognostic factors for decreased OS. Liver metastasis was significant independent prognostic factor for survival in univariate analysis, but not in multivariate analysis.

**TABLE 2:**
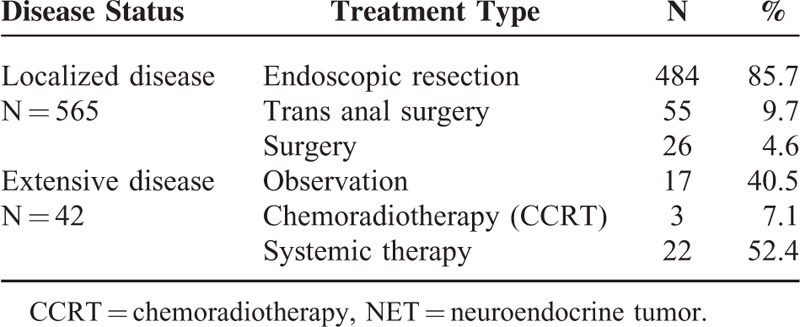
Treatment Patterns in 607 Hindgut NET Patients

### Systemic Therapies in 22 Hindgut NETs Patients

**FIGURE 3:**
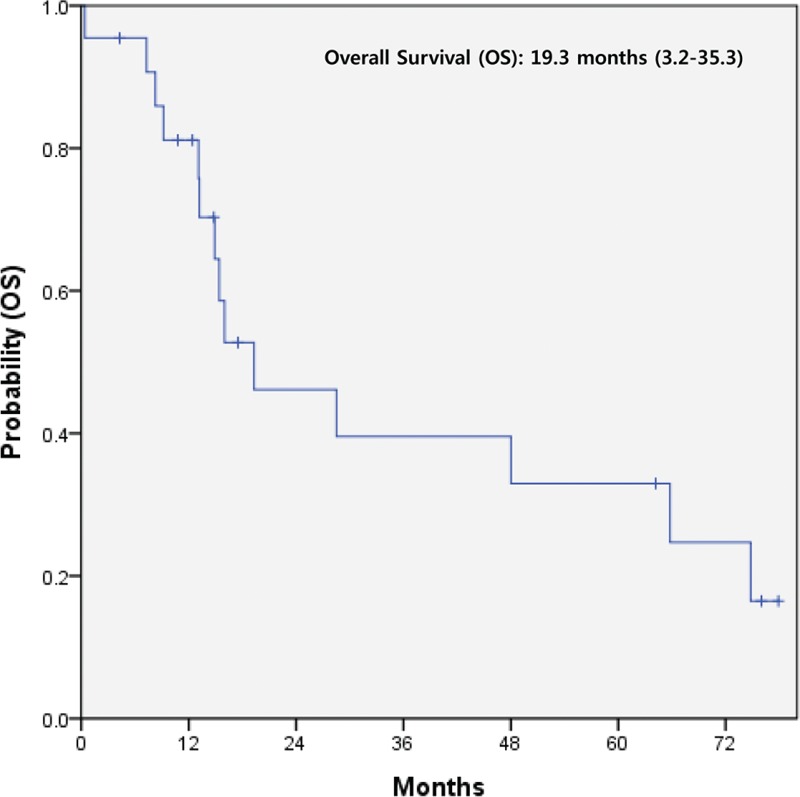
Kaplan–Meier curve of overall survival (OS) in 22 metastatic hindgut NET patients receiving systemic therapies. NET = neuroendocrine tumor, OS = overall survival.

Twenty-two of 42 patients with extensive disease received systemic therapy. Among these 22 patients, 12 patients received only first-line therapy, 8 had second-line, and only 2 patients had third-line (Table 3). As first-line chemotherapy, the most commonly used regimens were etoposide plus cisplatin (EP) (N = 7) and somatostatin analog (N = 7) followed by pazopanib (N = 3), etoposide, ifosfamide plus cisplatin (VIP) (N = 2), fluorouracil (FU) (N = 1), FU plus cisplatin (N = 1), and FU plus streptozocin (N = 1). During treatment courses, the most commonly used regimen was somatosatin analog. The median overall survival (OS) was 19.3 months (95% CI, 3.2–35.3) for 22 metastatic NET patients who underwent systemic therapy (Figure 3).

**TABLE 3:**
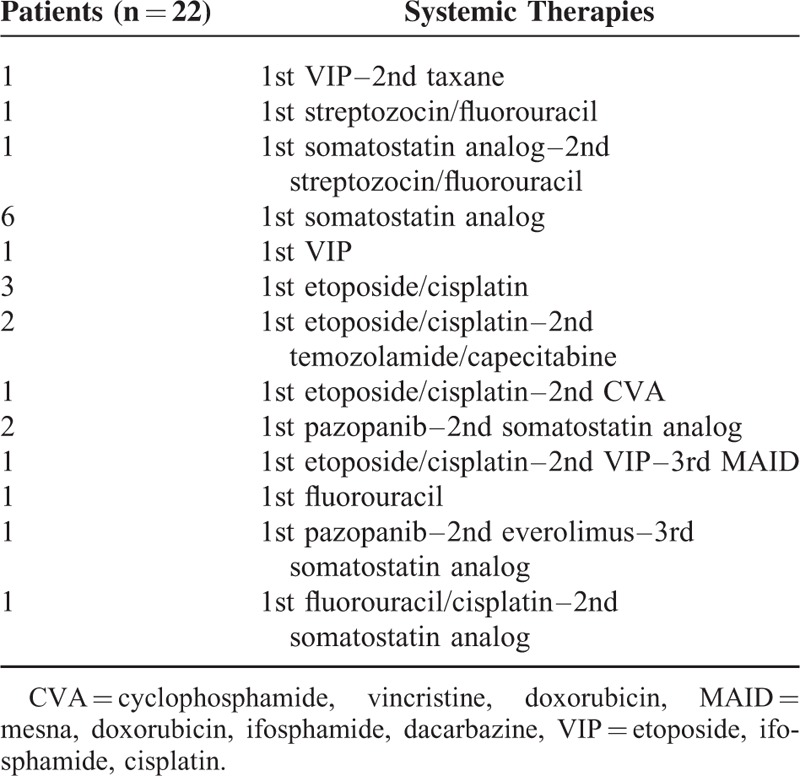
The Chemotherapy Regimens for 22 Hindgut NET Patients Receiving Systemic Therapy

## DISCUSSION

Our study demonstrated clinicopathologic features, treatment patterns, and survival according to disease extent in 607 hindgut NETs. There have been few studies of the characteristics, treatment, and survival of colorectal (CRC) NETs. In 2011, a Colonoscopy Study Group of the Korean Society of Coloproctology described the clinical characteristics of CRC carcinoid tumors.^9^ However, they did not analyze survival, classify patients using the 2010 WHO classification system or administer systemic treatment. Our findings will be useful as a foundation for further research into hindgut NETs.

The primary tumor sites in this study were the rectum (86.7%) and colon (13.3%) (rectum to colon ratio of 6.5 to 1). This finding was consistent with that from a previous study.^9^ The rectum to colon ratio was 6.0 to 1.0 in data from The Gastrointestinal Pathology Study Group of Korean Society of Pathologists. However, the ratio of rectum to colon was 34.7 to 1.0 in another Korean study.^5^ In a Japanese study for CRC NETs, the ratio of rectum to colon was 12.6 to 1.0.^10^ This discrepancy in the ratio of rectum to colon may be caused by the heterogeneity of the patient population in this study. According to the 2010 WHO classification, 578 of 607 (95.2%) had grade I NETs. Grade 3 NECs were diagnosed in only 12 patients (2.0%) and the colon was the primary site in 10 of 12 NEC patients (83.3%).

We identified only 3 (0.5%) hindgut NET patients with hormonal symptoms such as diarrhea, facial flushing, and sweating. These 3 patients had extensive disease with liver metastasis. This finding was concordant with findings from our prior report.^11^ Previously, our group revealed 4 (0.9%) of 470 patients had foregut, midgut, and hindgut NETs. However, several studies have reported that >30% of NET patients present with endocrine symptoms.^12–14^ The difference between our studies and those of other groups is that nonfunctional NET occurs more frequently in Koreans, or the widespread use of endoscopy and the development of more sensitive diagnostic tools has resulted in the detection of small NET at an early stage. However, we conducted the effect of surgery/treatment type on 5/10-year survival of 565 Hindgut NETs patients with localized disease. For NET patients with localized disease, the choice between endoscopic resection and (transanal) surgery was usually determined based on tumor size. In our institution, NETs of <1 cm usually managed by endoscopic procedure and NETs of >1 cm were treated by (transanal) surgery. Our analysis revealed that there was no significant difference of 5/10-year survival of 565 Hindgut NETs Patients with localized disease according to surgery/treatment type. However, our finding must be interpreted with caution. This analysis was retrospectively conducted, and the number of recurrence and death in patients with localized disease was too small.

The effects of systemic therapies in metastatic hindgut NETs or NEC have not been clearly established. The clinical behavior of grade 3 GEP-NEC is similar to that of small cell lung cancer (SCLC), which is known to be responsive to etoposide and cisplatin (EP).^15^ Thus, irrespective of primary sites such as foregut, midgut, and hindgut, EP has been used as a reference treatment for NEC. The PROMID study and CLARINET confirmed the antitumor effect of the somatostatin analog in functional and/or nonfunctional GEP-NETs.^16,17^ Moreover, 2 agents inhibiting relevant molecular targets have been approved by the U.S. Food and Drug Administration (FDA) for NET with promising outcomes.^18,19^ However, these specific therapeutic challenges have not focused on hindgut NET with G1 or G2 based on the 2010 WHO classification. Thus, currently, systemic therapies for hindgut NET vary among individual physicians. In our study, 22 metastatic hindgut NET or NEC patients received palliative systemic treatments. As a first-line therapy, 10 patients (45.4%) were treated by EP or VIP regimen. Twelve patients (54.5%) received somatostatin analog during the treatment course. Hindgut NETs with extensive disease have been managed according to the guidance extrapolated from trials with foregut or midgut NETs.

In this study, distant metastases showed a low rate of 6.9% (42 of 607) compared with other reports.^12,14^ Among 42 patients, there were 7 grade III NEC patients. Of all 12 grade III NEC patients, 58.3% (7 of 12) had distant metastasis at diagnosis. For 22 metastatic NET patients with systemic therapy, the overall survival (OS) was 19.3 months (95% CI, 3.2–35.3). Among these patients, the subpopulation with grade III NEC showed a median OS of 14.9 months (95% CI, 4.8–24.9). Our survival data for grade III hindgut NEC were consistent with that from a previous study for GEP- and hepatobiliary NEC.^20^

Our study was a retrospective analysis of patients with heterogeneous characteristics. In addition, there was a lack of samples for extensive diseases and systemic therapies. Nevertheless, this analysis demonstrates the importance of identifying clinicopathologic features, determining treatments and assessing survival for hindgut NET patients. Further studies are needed to understand the biologic behavior and to guide the systemic therapy for hindgut NET and NEC.
